# Predictive Modelling Based on Statistical Learning in Biomedicine

**DOI:** 10.1155/2017/4041736

**Published:** 2017-09-28

**Authors:** Olaf Gefeller, Benjamin Hofner, Andreas Mayr, Elisabeth Waldmann

**Affiliations:** ^1^Department of Medical Informatics, Biometry & Epidemiology, Friedrich-Alexander University Erlangen-Nürnberg, Waldstr. 6, 91054 Erlangen, Germany; ^2^Section Biostatistics, Paul-Ehrlich-Institut, Langen, Germany; ^3^Department of Statistics, Ludwig-Maximilians-Universität München, Munich, Germany

Twenty years ago, the journal Computational and Mathematical Methods in Medicine was launched under its previous title Journal of Theoretical Medicine. During those years at the end of the last century, the understanding of machine learning technology and its potential combination with statistical modelling approaches was in its infancy. The modern term “statistical learning” for this fusion of methodology from different scientific areas could already be found in the scientific literature (see Vapnik [1, 2]), but its meaning was slightly different from today. The famous textbook by Hastie et al. [3] popularised the term in its current meaning when being published in its first edition in 2001. During recent years, considerable research has been devoted to exploring this combination of state-of-the-art statistical methodology with machine learning techniques. Such an approach provides many practical advantages, particularly regarding data situations frequently encountered in modern biomedical research characterized by large numbers of potential features or variables. In such situations, the primary aim is often to obtain sparse and explanatory models, which can be generalized effectively. Via statistical learning approaches, interpretable prediction rules leading to accurate forecasts for future or unseen observations can be deduced from potentially high-dimensional data.

This special issue is devoted to this evolving research area at the intersection between different scientific branches. It attracted a broad spectrum of methodological contributions regarding different types of algorithms and fields of biomedical application. Out of sixteen submissions that were rigorously evaluated by international experts, nine made it into this issue. The compilation of papers in this special issue consists of one review paper and eight original research articles.

In their review titled “An Update on Statistical Boosting in Biomedicine” A. Mayr et al. give an overview of recent developments in the evolving area of statistical boosting algorithms. In doing so, they update and expand earlier reviews of this specific area of statistical learning research [4–6]. For the first time, recent methodologic research on boosting functional data and on the application of boosting techniques in advanced survival modelling is reviewed. Modern biomedical applications of this type of statistical learning are also sketched to provide an overview not only of recent methodologic improvements but also of practical implementation of boosting in answering biomedical research questions.

In the paper titled “A Multicriteria Approach to Find Predictive and Sparse Models with Stable Feature Selection for High-Dimensional Data” A. Bommert et al. propose a way to select models based on multiple important criteria: prediction accuracy as well as sparsity and stability of the model. For model stability, the authors investigate, analytically and in a simulation study, various stability measures and conclude that the Pearson correlation has the best properties. In another simulation study with various learning approaches such as random forests, support vector machines, lasso regression, and boosting in combination with a variety of filter methods preselecting features, they investigate Pareto fronts and conclude that it is possible to find models with a stable selection of only a few features without losing much predictive accuracy.

The paper titled “Correcting Classifiers for Sample Selection Bias in Two-Phase Case-Control Studies” by N. Krautenbacher et al. aims at improving results of models based on stratified data. It gives a very detailed explanation of the general problem of the resulting selecting bias when aiming at capturing more information by using a higher proportion of individuals with rare outcomes and a thorough summary of existing methods. Furthermore, two novel approaches are presented which outperform the state-of-the-art methods when being used in the random forests context and perform equally well when being used for logistic regression.

The paper titled “Integration of Multiple Genomic Data Sources in a Bayesian Cox Model for Variable Selection and Prediction” by T. Treppmann et al. is the only Bayesian contribution to the special issue. The authors integrate information from different sources to improve variable selection performance and prediction ability in the context of high-dimensional survival analysis. In order to achieve their goal, they combine Lee et al.'s approach [7] with George and McCulloch's Gibbs sampler [8]. Basically, the latter approach allows variable-specific penalties for the lasso-type approach of the former. In their biomedical application, the authors use information from copy number variation data to improve a model based on gene expressions.

In their paper titled “Pathway-Based Kernel Boosting for the Analysis of Genome-Wide Association Studies” S. Friedrichs et al. describe a framework to incorporate genetic pathways, that is, gene-interaction networks, in prediction models for the analysis of genome-wide association studies (GWAS). The approach adapts a boosting method with specific kernel-based learners. The authors show that their approach identifies important genetic factors while evading the issue of multiple testing. As the genetic interaction networks can be biologically interpreted, the approach facilitates understanding the biological processes involved in disease susceptibility. Furthermore, it enables the prediction of clinical outcomes for new patients and thus constitutes a powerful tool in the analysis of GWAS data.

The article titled “Probing for Sparse and Fast Variable Selection with Model-Based Boosting” by J. Thomas et al. proposes a fundamentally new concept to select the optimal number of iterations for statistical boosting algorithms. This so-called stopping iteration is the main tuning parameter for these kinds of algorithms and represents the classical trade-off between variance and bias. Typically it is selected based on resampling procedures focusing on the predictive risk and therefore on prediction accuracy. The authors propose focusing on the variable selection properties of the algorithm: they incorporate additional noninformative probes (shadow variables) for each candidate variable and stop the algorithm once the first of these probes was selected. This new approach is considerably faster than resampling, because the model is fitted only once without additional tuning. In large-scale simulations, the authors show that their approach leads to sparser models with less false positives than traditional methods to determine the stopping iteration.

The focus of the paper titled “Nonparametric Subgroup Identification by PRIM and CART: A Simulation and Application Study” by A. Ott and A. Hapfelmeier lies on traditional machine learning technology. Classification and Regression Trees (CART) have been introduced more than 30 years ago by Breiman et al. [9] and have made their way into the standard repertoire of methods to identify homogenous subgroups in high-dimensional data situations. The Patient Rule Induction Method (PRIM), which has been developed for the same purpose in biomedical applications based on a computational idea of Friedman and Fisher [10], has attracted some interest but is less often used in practice. Ott and Hapfelmeier compare the two strategies by means of an exhaustive simulation study. In particular, they show in which scenarios PRIM outperforms CART. The manuscript covers also an application using a clinical data set in which the two approaches produce similar results. However, the authors also demonstrate in their application that CART, although simpler to implement, is a rather static technique, whereas PRIM can be flexibly tuned by the user.

In their paper titled “IPF-LASSO: Integrative* L*_1_-Penalized Regression with Penalty Factors for Prediction Based on Multi-Omics Data” A.-L. Boulesteix et al. focus on the problem of integrating high-dimensional molecular, genetic, or other “omics” data from different sources or modalities together with clinical variables into one prediction model. They adapt the classical lasso (which leads often to very similar solutions to statistical boosting approaches; see Hepp et al. [11]) by introducing different* L*_1_ penalties for the modalities in order to account for their different importance. The application of the approach is illustrated via the development of prediction models for the survival of cancer patients based on clinical variables, microarray gene expressions, and somatic copy number alterations.

In their interesting application-oriented paper titled “Dysphonic Voice Pattern Analysis of Patients in Parkinson's Disease Using Minimum Interclass Probability Risk Feature Selection and Bagging Ensemble Learning Methods” Y. Wu et al. compare different machine learning approaches in their discriminatory performance on voice pattern data from patients with Parkinson's disease and healthy controls. Their novel contribution consists of suggesting a new method of feature selection from voice patterns subsequently processed by machine learning algorithms. Their results show superiority of classification performance of their approach termed “interclass probability risk method” over traditional competitors.
